# Sex Differences in Baseline Characteristics and Long-Term Outcomes of Primary Glomerular Diseases: Insights from the TSN-GOLD Registry

**DOI:** 10.3390/jcm15114017

**Published:** 2026-05-22

**Authors:** Dilek Guven Taymez, Ece Uk, Necmi Eren, Ahmet Alper Kıykım, Mevlut Tamer Dincer, Musa Pınar, Sim Kutlay, Tugba Elif Ozler, Erhan Tatar, Halime Soyak Kabaca, Taner Basturk, Onur Tunca, Ezgi Coskun Yenigun, Dilek Torun, Kultigin Turkmen, Melike Betul Ogutmen, Serap Yadigar, Ozkan Gungor, Gulizar Sahin, Mehmet Deniz Aylı, Ilhan Kurultak, Meltem Gursu, Ozant Helvacı, Mehmet Tanrısev, Nurhan Bilen, Erkan Sengul, Nedim Yılmaz Selcuk, Nimet Aktas, Arzu Özdemir, Zeki Kemec, Düriye Deren Oygar, Murat Duranay, Zeki Aydın, Sabahat Alısır Ecder, Alper Azak, Bulent Kaya, Metin Ergul, Ahmet Burak Dirim, Serap Demir, Seyda Gul Ozcan, Hamad Dheir, Engin Onan, Gizem Kumru, Savas Ozturk

**Affiliations:** 1 Department of Nephrology, Kocaeli City Hospital, 41050 Kocaeli, Turkey; 2 Department of Nephrology, İstanbul Faculty of Medicine, İstanbul University, 34010 İstanbul, Turkey; eceukdr@gmail.com (E.U.); ahmetburakdirim@gmail.com (A.B.D.); savasozturkdr@gmail.com (S.O.); 3 Department of Nephrology, Faculty of Medicine, Kocaeli University, 41380 Kocaeli, Turkey; necmieren.kou@gmail.com (N.E.); dr.metinergul@gmail.com (M.E.); 4 Department of Internal Medicine, Division of Nephrology, Mersin University School of Medicine, 33110 Mersin, Turkey; ahmetkiykim@gmail.com (A.A.K.); serapbas@yahoo.com (S.D.); 5 Department of Internal Medicine, Division of Nephrology, Cerrahpasa Faculty of Medicine, Istanbul University, 34360 Istanbul, Turkey; mevluttamer.dincer@iuc.edu.tr (M.T.D.); seydagulozcan@gmail.com (S.G.O.); 6 Department of Nephrology, Faculty of Medicine, Sakarya University, 54020 Sakarya, Turkey; musapinar43@hotmail.com (M.P.); hamaddheir@sakarya.edu.tr (H.D.); 7 Department of Nephrology, İbni Sina Hospital, Ankara University, 06100 Ankara, Turkey; skutlay@hotmail.com (S.K.); gkumru@ankara.edu.tr (G.K.); 8 Division of Nephrology, Haseki Training and Research Hospital, 41050 İstanbul, Turkey; telifsenel@gmail.com; 9 Department of Nephrology, Medical Point Hospital, Izmir Economy University, 35575 İzmir, Turkey; etatar@hotmail.com; 10 Department of Internal Medicine, Division of Nephrology, Faculty of Medicine, Bursa Uludağ University, 16059 Bursa, Turkey; halimesoyak@uludag.edu.tr; 11 Department of Nephrology, Sisli Hamidiye Etfal Training and Research Hospital, University of Health Sciences, 34371 Istanbul, Turkey; tanerbast@yahoo.com; 12 Department of Nephrology, Faculty of Medicine, Afyonkarahisar Health Sciences University, 03030 Afyonkarahisar, Turkey; dronurtunca@hotmail.com; 13 Department of Nephrology, Ankara Bilkent City Hospital, 06800 Ankara, Turkey; drezgi_76@hotmail.com; 14 Department of Nephrology, Dr. Turgut Noyan Training and Research Hospital, Baskent University, 01250 Adana, Turkey; dilektorun@hotmail.com (D.T.); onanmd@gmail.com (E.O.); 15 Department of Nephrology, Faculty of Medicine, Necmettin Erbakan University, 42090 Konya, Turkey; ucmdkt@gmail.com; 16 Haydarpasa Numune Training and Research Hospital, University of Health Sciences, 34668 Istanbul, Turkey; betulogutmen@gmail.com; 17 Department of Nephrology, Kartal Training and Research Hospital, 34865 İstanbul, Turkey; drserapyadigar@gmail.com; 18 Department of Nephrology, Faculty of Medicine, Kahramanmaras Sutcu Imam University, 46050 Kahramanmaras, Turkey; ozkan.gungor@yahoo.com; 19 Department of Nephrology, Sultan Abdulhamid Han Research and Training Hospital, University of Health Sciences, 34668 Istanbul, Turkey; gulimanga@yahoo.com; 20 Department of Nephrology, University of Health Sciences, Etlik City Hospital, 06010 Ankara, Turkey; d_ayli@hotmail.com; 21 Department of Nephrology, Trakya University Faculty of Medicine, Balkan Campus, 22030 Edirne, Turkey; ilhankurultak@yahoo.com; 22 Department of Nephrology, Faculty of Medicine, Bezmialem Vakıf University, 34093 Istanbul, Turkey; meltem1401@yahoo.com; 23 Department of Internal Medicine, Division of Nephrology, Gazi University Faculty of Medicine, 06560 Ankara, Turkey; 24 Department of Nephrology, Faculty of Medicine, University of Health Sciences, Tepecik Training and Research Hospital, 35120 Izmir, Turkey; mehmettanrisev@gmail.com; 25 Department of Nephrology, Bursa Faculty of Medicine, Bursa City Hospital, University of Health Sciences, 16110 Bursa, Turkey; nurhan-topal@hotmail.com; 26 Department of Nephrology, Hamidiye Faculty of Medicine, Kocaeli City Hospital, University of Health Sciences, 41700 Kocaeli, Turkey; dr.erkansengul@hotmail.com; 27 Department of Nephrology, Department of Internal Medicine, Meram Faculty of Medicine, Necmettin Erbakan University, 42080 Konya, Turkey; nyselcuk@yahoo.com; 28 Department of Nephrology, Bursa Yuksek Ihtisas Training and Research Hospital, University of Health Sciences, 16310 Bursa, Turkey; nimetaktas@gmail.com; 29 Bakirkoy Dr. Sadi Konuk Training and Research Hospital, University of Health Sciences, 34147 Istanbul, Turkey; arzukayalar@yahoo.com; 30 Division of Nephrology, Department of Internal Medicine, Batman Training and Research Hospital, 72070 Batman, Turkey; zekikemec@gmail.com; 31 Division of Nephrology, Department of Internal Medicine, Dr. Burhan Nalbantoglu State Hospital, 99010 Lefkosa, Cyprus; derenoygar@yahoo.com; 32 Department of Nephrology, Ankara Training and Research Hospital, 06230 Ankara, Turkey; duranaymurat@hotmail.com; 33 Darıca Farabi Training and Research Hospital, 41700 Darıca, Turkey; zekiaydindr@yahoo.com; 34 Division of Nephrology, Istanbul Medeniyet University, 41050 İstanbul, Turkey; sabahatalisir@yahoo.com; 35 Department of Nephrology, Balikesir Ataturk City Hospital Training and Research Center, University of Health Sciences Turkiye, 10185 Balikesir, Turkey; dralperazak@gmail.com; 36 Department of Nephrology, Cukurova University Faculty of Medicine, 01330 Adana, Turkey; bulentkaya32@gmail.com

**Keywords:** primary glomerular disease, sex differences, chronic kidney disease, prognosis, biopsy, registry

## Abstract

**Background:** Primary glomerular diseases (PGDs) are a leading cause of end-stage kidney disease (ESKD). While sex differences in chronic kidney disease progression have been reported, their role in PGDs remains unclear. **Methods:** We analyzed 2081 patients with biopsy-proven PGDs from the Turkish Society of Nephrology Glomerular Diseases Working Group (TSN-GOLD) registry. Baseline demographic, clinical, biochemical, and histopathological characteristics were compared between women and men. Outcomes were assessed as a composite of ESKD or death. Logistic and Cox regression models were applied to identify independent risk factors. Kaplan–Meier analyses evaluated survival differences. **Results:** At baseline, women and men had similar rates of hypertension (36.8% vs. 34.7%, *p* = 0.322). Women more frequently presented with leukocyturia (31.7% vs. 17.2%, *p* < 0.001) and hematuria (57.8% vs. 52.3%, *p* = 0.016), whereas men had higher proteinuria (5011 ± 4925 vs. 4388 ± 4529 mg/day, *p* = 0.003) and were more likely to be active smokers (20.7% vs. 7.4%, *p* < 0.001). Serum albumin (3.3 ± 0.8 vs. 3.2 ± 0.9 g/dL) and eGFR (79.7 ± 44.3 vs. 78.7 ± 45.5 mL/min/1.73 m^2^) were comparable between sexes (both NS). During a median follow-up of 24 months (IQR 7-60), 431 patients (20.7%) reached the composite outcome of ESKD or death (137 deaths [6.6%], 294 ESKD [14.1%]). In the multivariable Cox regression model, lower baseline eGFR (HR 0.98, 95% CI 0.98–0.98, *p* < 0.001), lower serum albumin (HR 0.65, 95% CI 0.55–0.77, *p* < 0.001), higher proteinuria (HR 1.03, 95% CI 1.00–1.05, *p* = 0.043), and biopsy diagnosis of RPGN (HR 3.78, 95% CI 1.37–10.45, *p* = 0.010) were independent predictors of poor prognosis. Sex was not an independent predictor of outcome (*p* = 0.48). Kaplan–Meier analysis demonstrated no significant survival difference between women and men (log-rank *p* = 0.052). **Conclusions:** In this nationwide PGD cohort, women and men differed significantly in baseline biochemical and clinical parameters, yet sex was not independently associated with progression to ESKD or death. Instead, disease severity at baseline and histopathological features were the main drivers of prognosis.

## 1. Introduction

Glomerular diseases remain a leading cause of chronic kidney disease (CKD) and end-stage kidney disease (ESKD) worldwide, imposing a substantial burden on patients and healthcare systems. Recent epidemiologic data suggest that glomerulopathies account for up to 20–25% of incident ESKD cases globally [1]. Despite advances in diagnostic techniques and therapeutic strategies, long-term prognosis varies widely across different histopathological subtypes.

Sex-related differences in kidney disease progression have been reported across both CKD and primary glomerulopathies. Biological factors such as sex hormones, genetic background, and immune response are believed to modulate disease onset, progression, and treatment response. For instance, several studies have demonstrated that men may experience more rapid kidney function decline compared to women, particularly in IgA nephropathy (IgAN) and focal segmental glomerulosclerosis (FSGS) [2]. Conversely, women have been reported to have higher prevalence of autoimmune-mediated glomerular diseases, such as lupus nephritis, although outcomes may not always be superior [3].

Large cohort studies have emphasized the prognostic importance of baseline kidney function, proteinuria, and histopathological markers including tubular atrophy, interstitial fibrosis, and crescents, which remain strong predictors of renal survival independent of clinical factors [4]. Yet, the interaction between sex and these established risk factors remains underexplored across a broad spectrum of biopsy-proven glomerular diseases [5].

Given these gaps, we analyzed a large multicenter cohort of 2081 patients with biopsy-proven glomerular diseases, aiming to (i) characterize sex-specific differences in clinical, biochemical, and histopathological features at baseline; and (ii) evaluate the impact of sex on renal outcomes, including progression to ESKD and mortality.

## 2. Methods

### 2.1. Study Design and Data Source

This study was conducted using data from the Turkish Society of Nephrology Glomerular Diseases Study Group (TSN-GOLD) registry, which is a nationwide, multicenter, retrospective, observational registry. The registry was established in 2009 and involves more than 40 nephrology centers distributed across Turkey. Its main purpose is to collect standardized clinical, laboratory, and histopathological data of patients with biopsy-proven glomerular diseases. The methodology of the registry has been described previously in detail.

Data are collected using standardized electronic case report forms (CRFs) by trained nephrologists at each participating center. The registry includes demographic variables, comorbid conditions, baseline and follow-up biochemical results, detailed histopathological findings, treatment regimens, and clinical outcomes. Quality checks and periodic audits are performed by the central coordinating committee to ensure data accuracy and completeness.

### 2.2. Study Population

Between May 2009 and May 2022, a total of 7485 patients with kidney biopsies were registered from 59 nephrology centers participating in the TSN-GOLD registry. After excluding patients with secondary glomerular diseases (e.g., lupus nephritis, vasculitis, amyloidosis, diabetic nephropathy) and those lacking adequate light microscopy and immunofluorescence (IF) findings, 6472 patients with biopsy-proven primary glomerular diseases were eligible for further evaluation. Patients with primary systemic ANCA-associated vasculitis were excluded. However, isolated ANCA seropositivity without clinical or histopathological evidence of systemic vasculitis was not considered an exclusion criterion.

This study was not restricted to patients with nephrotic syndrome but included a broader cohort of patients with biopsy-proven glomerular diseases. The study population and eligibility criteria were clearly defined in the Methods section, including prespecified thresholds for eGFR and proteinuria. Patients with incomplete clinical data or inadequate follow-up were excluded from the analysis.

Of these, 65 patients with acute post-infectious glomerulonephritis, 189 patients with rare or unclassified glomerular pathologies, and 3 patients with missing sex information were excluded. Additionally, 4134 patients were excluded due to incomplete outcome data or insufficient follow-up information. After applying these criteria, a total of 2081 patients with complete clinical, histopathological, and outcome data were included in the final analysis. This study was not restricted to patients with nephrotic syndrome but included a broader cohort of patients with biopsy-proven glomerular diseases. No predefined eGFR or proteinuria cutoff values were used as inclusion criteria for registry enrollment.

The MEST-C score was evaluated and reported only for patients with IgA nephropathy and was not applied to other glomerular diseases.

The final cohort consisted of 2081 patients after exclusion of patients with missing outcome information. Baseline characteristics were defined at the time of renal biopsy. Follow-up data were collected annually during routine clinical visits or at the time of clinical events (remission, relapse, dialysis initiation, transplantation, or death).

## 3. Definitions

- **Composite outcome:** Defined as either progression to end-stage kidney disease (ESKD), indicated by initiation of chronic dialysis or kidney transplantation, or all-cause mortality during follow-up.
- **ESKD:** Defined as initiation of hemodialysis, peritoneal dialysis, or kidney transplantation.
- **Remission:** Complete remission was defined as proteinuria < 0.3 g/day with stable renal function, and partial remission was defined as ≥50% reduction in proteinuria from baseline to <3.5 g/day with stable renal function, consistent with KDIGO definitions [2].
- **Relapse:** Recurrence of proteinuria > 3.5 g/day or >50% increase from nadir values after partial or complete remission.
- **Hypertension:** Defined as documented diagnosis prior to biopsy or use of antihypertensive medications.
- **Diabetes mellitus:** Defined as physician diagnosis, use of antidiabetic medications, or fasting plasma glucose ≥ 126 mg/dL.
- **Leukocyturia** was defined as the presence of ≥5 leukocytes per high-power field on urine microscopy. Due to the retrospective multicenter design of the registry, urine culture data were not consistently available for all patients; therefore, differentiation between sterile leukocyturia and culture-confirmed urinary tract infection could not be uniformly established.

Histopathological variables were extracted from renal biopsy reports, including the number of glomeruli, percentage of global glomerulosclerosis, presence and type of crescents, interstitial fibrosis/tubular atrophy (IFTA), vascular changes, and immunofluorescence findings (IgA, IgM, IgG, C3, C1q, and light chains). These data were not centrally reviewed.

## 4. Statistical Analysis

Baseline clinical, biochemical, and histopathological characteristics were summarized as means ± standard deviation (SD) for normally distributed continuous variables, medians with interquartile range (IQR) for skewed variables, and frequencies with percentages for categorical variables. Between-group comparisons (e.g., male vs. female, active follow-up vs. composite outcome) were performed using the Student’s *t*-test or Mann–Whitney U test for continuous variables, and chi-square or Fisher’s exact test for categorical variables. Survival analyses were performed using the Kaplan–Meier method, and survival curves were compared with the log-rank test. Time-to-event was calculated from the date of renal biopsy to the occurrence of the composite outcome (ESKD or death), or censored at the last follow-up. Multivariable analyses were conducted to identify independent predictors of the composite outcome. Cox proportional hazards regression was applied for time-to-event analyses, and hazard ratios (HRs) with 95% confidence intervals (CIs) were reported. Variables with *p* < 0.10 in univariate analyses, as well as clinically relevant covariates (age, sex, baseline eGFR, biopsy diagnosis), were included in the multivariable model. Logistic regression was also performed for sensitivity analyses, with odds ratios (ORs) and 95% CIs reported. Model assumptions (linearity, proportional hazards) were checked before final analysis. A two-sided *p* < 0.05 was considered statistically significant. All analyses were performed using IBM SPSS Statistics for Windows (version 27.0, IBM Corp., Armonk, NY, USA).

## 5. Results

A total of 2081 patients with biopsy-proven primary glomerular diseases were included in the study, comprising 869 women (41.8%) and 1212 men (58.2%).

**Baseline characteristics:** Baseline demographic, biochemical, and clinical characteristics are presented in Table 1. The mean age was similar between women and men (42 ± 15 vs. 43 ± 16 years, *p* = 0.128). The prevalence of hypertension did not differ significantly (36.8% vs. 34.7%, *p* = 0.322). Women had a higher frequency of leukocyturia (31.7% vs. 17.2%, *p* < 0.001) and hematuria (57.8% vs. 52.3%, *p* = 0.016), while men had higher proteinuria (5011 ± 4925 vs. 4388 ± 4529 mg/day, *p* = 0.003) and were more likely to be active smokers (20.7% vs. 7.4%, *p* < 0.001). Serum albumin (3.3 ± 0.8 vs. 3.2 ± 0.9 g/dL) and eGFR (79.7 ± 44.3 vs. 78.7 ± 45.5 mL/min/1.73 m^2^) were comparable between sexes (both NS). Women had higher ESR (*p* = 0.046) and HDL-cholesterol (*p* < 0.001), whereas men had higher creatinine, urea, uric acid, blood pressure, body weight, and height (all *p* < 0.001).

### 5.1. Histopathological and Immunofluorescence Findings

Men more frequently had IgG (38.6% vs. 34.5%, *p* = 0.009) and IgA (38.3% vs. 35.1%, *p* = 0.026) positivity, while women showed higher IgM (34.5% vs. 27.6%, *p* = 0.019) and fibrinogen (9.5% vs. 6.6%, *p* = 0.021) deposition. Subepithelial deposits were more frequent in men (14.0% vs. 10.0%, *p* = 0.009) (Table 2).

### 5.2. Outcomes by Follow-Up Status

During a median follow-up of 24 months (IQR 7-60), 431 patients (20.7%) reached the composite outcome of ESKD or death (294 [14.1%] ESKD, 137 [6.6%] deaths). At the end of follow-up, 1650 patients (79.3%) remained in active follow-up, 294 patients (14.1%) had progressed to dialysis or transplantation, and 137 patients (6.6%) had died. Baseline clinical, biochemical, and demographic features stratified by outcome are presented in Table 3. Patients who developed ESKD or death were older, more likely to be hypertensive, and more frequently treated with immunosuppressive therapy. They showed significantly worse baseline kidney function (eGFR 46.6 vs. 87.5 mL/min/1.73 m^2^, *p* < 0.001), lower albumin (3.1 vs. 3.3 g/dL, *p* < 0.001), higher uric acid, and lower hemoglobin levels. Relapse, pretibial edema, leukocyturia, and hematuria were also more frequent among patients with poor outcomes. Importantly, low C3 levels (11.5% vs. 5.8%, *p* < 0.001) were more common among those with poor outcomes, and HBsAg positivity was also higher (3.4% vs. 2.8%, *p* = 0.009), whereas anti-HCV positivity was absent in the ESKD/death group (0% vs. 0.7%, *p* = 0.006). Proteinuria levels were similar between outcome groups. Differences in baseline histopathological features between groups are detailed in Table 4. Patients who developed ESKD or died exhibited more severe pathological changes, including higher rates of global and segmental sclerosis, endocapillary proliferation, interstitial fibrosis (≥25%), tubular atrophy (≥25%), and crescents (cellular and fibrocellular) (all *p* ≤ 0.005). Subendothelial deposits and vascular changes were also significantly more frequent (*p* < 0.01).

### 5.3. Multivariable Analyses

In the multivariable Cox proportional hazards model (Table 5), independent predictors of the composite outcome (ESKD or death) were biopsy diagnosis of RPGN (HR 3.78, 95% CI 1.37–10.45, *p* = 0.010), lower baseline eGFR (HR per mL/min/1.73 m^2^: 0.98, *p* < 0.001), lower serum albumin (HR per g/dL: 0.65, *p* < 0.001), and higher proteinuria (HR per g/day: 1.03, *p* = 0.043). Advanced interstitial fibrosis also showed a stepwise association with poor outcomes (Grade 2: HR 2.36, *p* = 0.004; Grade 3: HR 3.02, *p* = 0.009). Hematuria demonstrated a borderline protective effect (HR 0.76, *p* = 0.060). Neither age, sex, nor hypertension independently predicted outcomes.

Supplementary analyses confirmed these findings. In logistic regression (Supplementary Table S1), RPGN, MPGN, low albumin, low eGFR, and advanced tubular atrophy (Grade 3) were independent predictors. In the Cox model restricted to ESKD (excluding deaths) (Supplementary Table S2), independent predictors included RPGN, low eGFR, low albumin, high proteinuria, immunosuppressive treatment, and severe interstitial fibrosis (Grade 2–3), while hematuria remained protective. Similarly, logistic regression for ESKD alone (Supplementary Table S3) identified RPGN and MPGN as strong histopathological predictors, together with low eGFR, low albumin, and Grade 3 tubular atrophy.

Low complement (C3) levels and HBsAg positivity were associated with poorer unadjusted outcomes but did not retain significance after adjustment.

### 5.4. Survival Analysis

**Figure 1:**
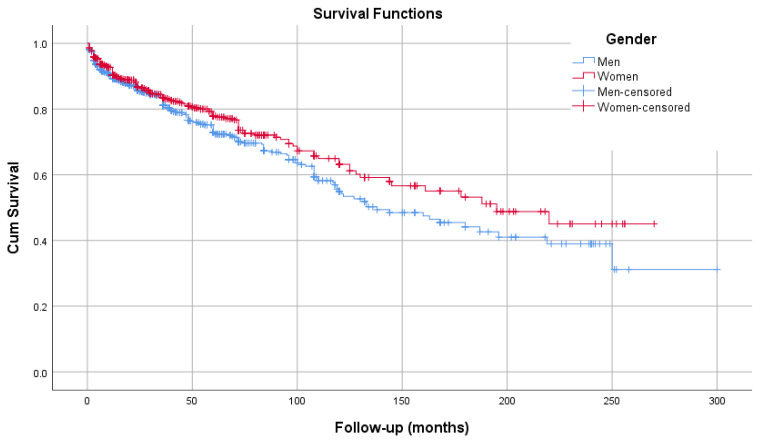
Kaplan–Meier survival curves for the composite outcome (ESKD or death) stratified by sex. (Kaplan–Meier renal survival curves for the composite renal outcome (ESKD or death) stratified by sex). Men are shown in blue and women in red. The projected median renal survival was 138 months for men and 195 months for women, based on Kaplan–Meier estimates. The median observational follow-up for the cohort was 24 months. Log-rank test: χ^2^ = 3.77, *p* = 0.052.

**Figure 2:**
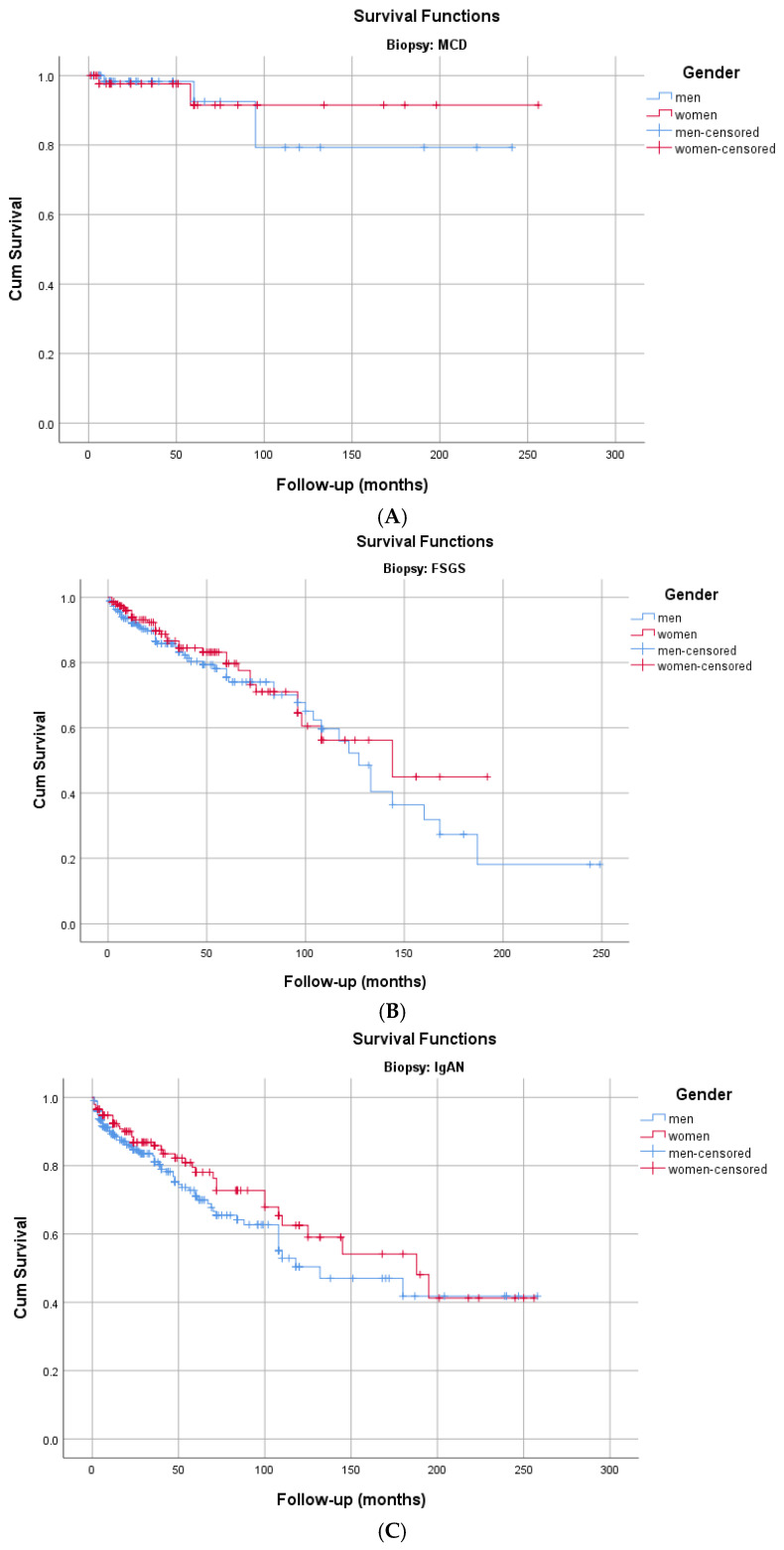

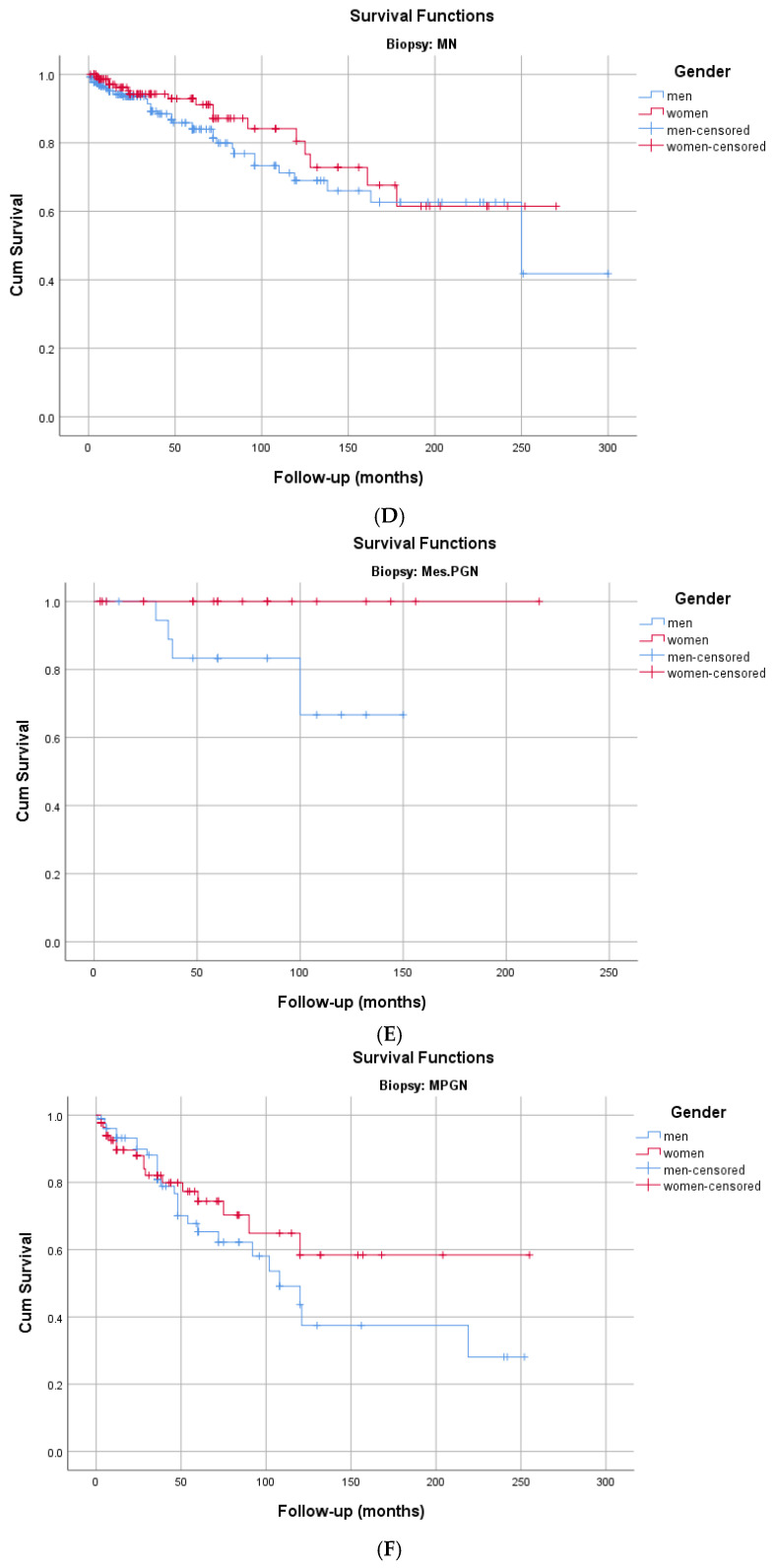

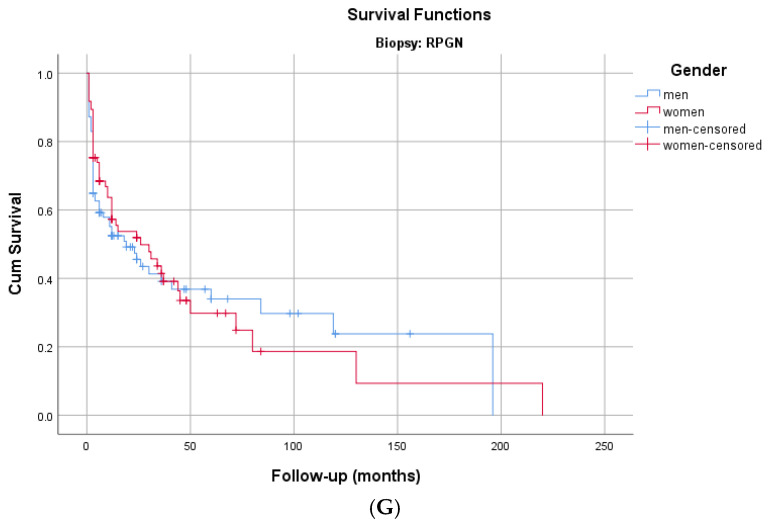
Kaplan–Meier survival curves by sex, stratified by biopsy diagnosis (Kaplan–Meier renal survival curves stratified by sex and biopsy diagnosis). No significant sex-related differences in renal survival were observed for minimal change disease (*p* = 0.837), FSGS (*p* = 0.409), IgA nephropathy (*p* = 0.153), membranous nephropathy (*p* = 0.176), MPGN (*p* = 0.318), or RPGN (*p* = 0.691). In contrast, women with mesangioproliferative glomerulonephritis had significantly better renal survival than men (*p* = 0.033). (**A**) Kaplan–Meier renal survival curves for patients with minimal change disease (MCD) stratified by sex. No significant sex-related difference in renal survival was observed between men and women (*p* = 0.837). (**B**) Kaplan–Meier renal survival curves for patients with focal segmental glomerulosclerosis (FSGS) stratified by sex. Renal survival did not significantly differ between men and women (*p* = 0.409). (**C**) Kaplan–Meier renal survival curves for patients with IgA nephropathy (IgAN) stratified by sex. No statistically significant sex-related difference in renal survival was observed (*p* = 0.153). (**D**) Kaplan–Meier renal survival curves for patients with membranous nephropathy (MN) stratified by sex. No significant sex-related difference in renal survival was observed between men and women (*p* = 0.176). (**E**) Kaplan–Meier renal survival curves for patients with mesangioproliferative glomerulonephritis (Mes.PGN) stratified by sex. Women demonstrated significantly better renal survival compared with men (*p* = 0.033). (**F**) Kaplan–Meier renal survival curves for patients with membranoproliferative glomerulonephritis (MPGN) stratified by sex. No statistically significant sex-related difference in renal survival was observed (*p* = 0.318). (**G**) Kaplan–Meier renal survival curves for patients with rapidly progressive glomerulonephritis (RPGN) stratified by sex. Renal survival did not significantly differ between men and women (*p* = 0.691).

Kaplan–Meier survival curves for the composite outcome (ESKD or death) by sex are shown in Figure 1. Median renal survival was longer in women (195 months) than in men (138 months), though the difference did not reach statistical significance (log-rank *p* = 0.052). Sex-stratified analyses by biopsy diagnosis are displayed in Figure 2. No significant sex differences were observed in survival among patients with minimal change disease, FSGS, IgA nephropathy, membranous nephropathy, MPGN, or RPGN. However, in mesangioproliferative GN, women demonstrated significantly better survival compared to men (log-rank *p* = 0.033).

## 6. Discussion

In this large nationwide registry of 2081 patients with biopsy-proven primary glomerular diseases (PGDs), we observed both biochemical and histopathological differences between men and women, whereas the distribution of PGD subtypes and biopsy indications did not differ significantly by sex. At baseline, women presented with lower serum creatinine, uric acid, hemoglobin, and hematocrit, but higher HDL cholesterol and serum albumin, compared with men. These findings are consistent with previously reported physiological sex-related differences in renal and metabolic parameters [6,7]. Histopathologically, men exhibited more frequent vascular changes and fibrous crescents, while women were more likely to demonstrate subepithelial immune deposits and IgG/IgA positivity on immunofluorescence [8]. Importantly, the relative frequencies of major GN subtypes, including focal segmental glomerulosclerosis (FSGS), IgA nephropathy, membranous nephropathy, and membranoproliferative GN (MPGN), were comparable between sexes, as were the clinical indications for kidney biopsy such as nephrotic versus nephritic presentations. These findings mirror European and North American registry studies, which also report no substantial sex-related variation in PGD subtype distribution [9,10].

When outcomes were considered, sex itself was not an independent predictor of the composite outcome of end-stage kidney disease (ESKD) or death in either logistic or Cox regression models. Instead, biopsy diagnosis (particularly RPGN and MPGN), lower baseline eGFR, hypoalbuminemia, and advanced tubular atrophy or interstitial fibrosis were consistent independent predictors of poor prognosis. These results emphasize the dominant role of disease severity and histopathological damage over sex in determining renal outcomes. Similar findings have been reported in Asian cohorts, where histologic chronicity indices, rather than sex, were associated with progression to ESKD [11]. The apparent lack of sex-specific effect on long-term outcomes is notable, especially in the context of baseline biochemical differences, and suggests that once adjusted for kidney function and histologic severity, sex-related biological variation does not independently influence prognosis.

Potential biological explanations for sex-related differences in PGDs likely involve complex interactions between sex hormones, immune regulation, and renal fibrotic pathways. Experimental studies have suggested that estrogens may exert anti-inflammatory and antifibrotic effects through modulation of transforming growth factor-β signaling, oxidative stress, and endothelial function, whereas androgens may promote glomerulosclerosis, intraglomerular hypertension, and tubulointerstitial fibrosis. In addition, sex-dependent differences in innate and adaptive immune responses may influence susceptibility to immune-mediated glomerular injury. Women generally exhibit stronger humoral and autoimmune responses, while men may demonstrate more pronounced profibrotic and hemodynamic progression pathways. Nevertheless, in our cohort, these biologically plausible sex-related mechanisms did not translate into independently different long-term renal outcomes after adjustment for baseline disease severity and histopathological damage.

Finally, the observation that the distribution of GN subtypes and biopsy indications did not differ significantly between men and women supports the notion that PGDs manifest with similar clinical triggers across sexes. Previous registry-based analyses from Europe and the United States have reported comparable results, where nephrotic syndrome remains the leading indication for biopsy across both sexes, followed by nephritic presentations [8]. Taken together, our findings provide novel insights into the interplay between sex, baseline clinical and pathological features, and renal prognosis in PGDs, highlighting that while sex influences presentation, it does not independently drive disease outcomes.

FSGS is one of the most common PGDs worldwide and an important cause of ESKD, particularly in younger patients. In our cohort, FSGS did not differ in frequency between sexes, and logistic regression suggested only a trend toward higher risk, not reaching statistical significance. Previous studies have shown conflicting data: while some cohorts reported male predominance and worse outcomes in men [12], others, particularly European registry analyses, found no sex-related difference in prognosis once proteinuria and histological chronicity were considered. Our findings support the latter, underscoring that disease severity rather than sex determines outcome in FSGS.

IgA nephropathy (IgAN) is another major PGD, with well-documented male predominance in Asian cohorts [13], but less pronounced sex differences in Western populations. In the present study, we observed no significant difference in biopsy-proven IgAN frequency between men and women, nor an independent prognostic role of sex in outcome models. Consistent with prior reports, lower baseline eGFR, albumin, and higher proteinuria remained the primary determinants of prognosis [14]. Interestingly, the protective association of hematuria against ESKD observed in Cox regression aligns with previous work suggesting that persistent hematuria may reflect preserved renal function rather than a pathogenic driver of progression [15].

In contrast, MPGN and RPGN were consistently associated with worse outcomes, independent of sex. Patients with RPGN, in particular, exhibited a markedly higher risk of progression to ESKD, consistent with its aggressive clinical course. This is in line with international registry data showing RPGN as one of the strongest predictors of poor renal survival across PGDs [16,17]. Similarly, MPGN, although less frequent, carried an increased risk of adverse outcome, echoing findings from the Italian Registry and USRDS analyses where MPGN patients had higher rates of kidney failure despite therapy [18]. Notably, these associations remained robust even after excluding deaths, highlighting the intrinsic aggressiveness of these entities.

The role of immunosuppressive therapy also warrants discussion. In logistic models including death, immunosuppression was not independently associated with outcomes, but in Cox regression excluding deaths, treatment was linked to increased risk of progression. This likely reflects treatment indication bias, where patients with more aggressive histological lesions are more likely to receive immunosuppression. Similar challenges in interpreting registry-based treatment-outcome associations have been reported in IgAN and FSGS cohorts [19,20,21], underscoring the need for carefully designed prospective trials.

The borderline “protective” effect of hematuria (HR 0.76, *p* = 0.06) is also consistent with previous studies suggesting that persistent hematuria often indicates earlier disease detection and preserved renal reserve, not a biologically protective process [6]. Finally, the positive association between global sclerosis and adverse outcomes (HR 1.01, *p* = 0.015) reinforces prior evidence that chronic histologic damage—rather than sex—drives prognosis in primary glomerular diseases [4,11].

## 7. Limitations

This study has several limitations. First, its retrospective design and reliance on registry data may have introduced reporting or selection bias. Second, missing data led to the exclusion of a proportion of patients, which may affect the generalizability of the results. Third, treatment protocols and follow-up practices varied between centers, potentially influencing outcomes. Because the registry included multiple glomerular disease subtypes, remission and relapse definitions were standardized primarily for cohort-level comparisons and may not fully reflect disease-specific criteria used in individual glomerulopathies such as IgA nephropathy.

Although HBsAg positivity was more frequent among patients with adverse outcomes in unadjusted analyses, this association did not remain significant after multivariable adjustment and should therefore be interpreted cautiously given the relatively low prevalence of hepatitis seropositivity and the limited follow-up duration. Finally, histopathological assessment was not centrally reviewed, and some heterogeneity in biopsy interpretation may exist. The TSN-GOLD registry uniquely enables histopathologic-outcome linkage across diverse PGD types in a national population—a capability lacking in most international registries.

## 8. Conclusions

In this multicenter TSN-GOLD registry study, sex was not independently associated with baseline severity or long-term outcomes in primary glomerular diseases. Women had slightly higher albumin and lower proteinuria, but these differences did not affect prognosis. Biopsy indications and GN subtype distribution were also similar by sex. Outcomes were mainly determined by histopathological severity, renal function, and diagnosis. RPGN and MPGN predicted the poorest survival, while advanced tubular atrophy, interstitial fibrosis, low albumin, reduced eGFR, and higher proteinuria were consistent risk factors. Thus, prognosis depends on disease subtype and pathology rather than sex, underscoring the value of large registries and the need for prospective trials in aggressive GN forms.

## Figures and Tables

**Table 1:** Baseline demographic, biochemical and clinical characteristics of patients (n = 2081).

Variable	Female (n = 869, 41.8%)	Male (n = 1212, 58.2%)	Total (n = 2081)	*p*-Value
Age (years), mean ± SD	42 ± 15	43 ± 16	43 ± 16	0.128
Pre-existing hypertension	319 (36.8%)	417 (34.7%)	736 (35.6%)	0.322
Diabetes mellitus (type 2)	100 (11.6%)	116 (9.7%)	216 (10.5%)	0.355
Biopsy indication (nephrotic syndrome)	423 (50.1%)	611 (51.9%)	1034 (51.1%)	0.101
Histopathology (MEST-C, etc.)	M1: 72.9% E1: 33.8% S1: 49.7% T1–T2: 56.5%	M1: 77.6% E1: 29.8% S1: 52.6% T1–T2: 56.3%	M1: 75.9% E1: 31.3% S1: 51.5% T1–T2: 56.4%	NS
Biochemistry (mean ± SD)				
Creatinine (mg/dL)	1.5 ± 1.7	1.8 ± 2.0	1.7 ± 1.9	<0.001 *
eGFR (ml/min/1.73 m^2^, CKD-EPI 2021	79.7 ± 44.3	78.7 ± 45.5	79.1 ± 45.0	NS
Urea/BUN (mg/dL)	52 ± 43	60 ± 48	56 ± 46	<0.001 *
Uric acid (mg/dL)	5.9 ± 1.9	6.8 ± 1.9	6.4 ± 2.0	<0.001 *
Triglycerides (mg/dL)	192 ± 115	217 ± 140	207 ± 131	<0.001 *
HDL (mg/dL)	55 ± 23	48 ± 18	51 ± 20	<0.001 *
LDL (mg/dL)	163 ± 76	166 ± 84	165 ± 81	NS
Hemoglobin (g/dL)	11.8 ± 2.0	13.2 ± 2.5	12.6 ± 2.4	<0.001 *
Hematocrit (%)	36 ± 5	39 ± 7	38 ± 7	<0.001 *
Proteinuria (mg/day)	4388 ± 4529	5011 ± 4925	4751 ± 4773	0.003 *
Sedimentation (mm/h)	45 ± 29	41 ± 32	43 ± 31	0.046 *
Blood pressure SBP/DBP (mmHg)	131 ± 20/82 ± 11	135 ± 21/83 ± 12	133 ± 20/83 ± 12	<0.001 *
Weight (kg)	71 ± 16	79 ± 14	76 ± 16	<0.001 *
Height (cm)	159 ± 16	172 ± 14	167 ± 16	<0.001 *
Follow-up (months)	24 (7–60)	24 (7–60)	24 (7–60)	NS
Immunosuppressive treatment, n (%)	520 (62.4%)	763 (66.6%)	1283 (64.8%)	0.049 *
First immunosuppressive response (Remission), n (%)	311 (67.9%)	440 (62.8%)	751 (64.8%)	0.073
Remission type, Partial/Complete: n (%)	160 (52.1%)/147 (47.9%)	246 (56.4%)/190 (43.6%)	406 (54.6%)/337 (45.4%)	0.246
Relapse, n (%)	98 (34.3%)	149 (35.6%)	247 (35.0%)	0.723
Mortality	57 (6.6%)	80 (6.6%)	137 (6.6%)	0.970
Composite outcome (Ex or KRT), n (%)	164 (18.9)	267(22.0)	431(20.7)	0.08
Still active follow-up, n (%)	705 (81.1%)	945 (78.0%)	1650 (79.3%)	0.128
Dialysis or transplantation, n (%)	107 (12.3%)	187 (15.4%)	294 (14.1%)	0.128

* *p* < 0.05, Abbreviations: MEST-C, Mesangial hypercellularity, Endocapillary hypercellularity, Segmental glomerulosclerosis, Tubular atrophy/interstitial fibrosis, and Crescents score; eGFR, estimated glomerular filtration rate; BUN, blood urea nitrogen; HDL, high-density lipoprotein; LDL, low-density lipoprotein; SBP/DBP, systolic blood pressure/diastolic blood pressure; KRT, kidney replacement therapy. NS: nephrotic syndrome,* Statistically significant difference (*p* < 0.05).

**Table 2:** Histopathologic and immunofluorescence findings.

Variable	Female (n = 869)	Male (n = 1212)	Total (n = 2081)	*p*-Value
Total glomeruli (median, IQR)	15 (10–24)	15 (10–22)	15 (10–23)	ns
Globally sclerotic glomeruli (median, IQR)	1 (0–4)	1 (0–4)	1 (0–4)	ns
Cellular crescents (median, IQR)	0 (0–0)	0 (0–0)	0 (0–0)	ns
Fibrocellular crescents (median, IQR)	0 (0–0)	0 (0–0)	0 (0–0)	**0.049**
Fibrous crescents (median, IQR)	0 (0–0)	0 (0–0)	0 (0–0)	**0.025**
Mesangial proliferation present	485 (58.2%)	632 (54.5%)	1117 (56.0%)	ns

Abbreviations: ns, not significant; IQP, interquartile percentage.

**Table 3:** Baseline clinical, biochemical and demographic characteristics by outcome.

Variable	Active Follow-Up (n = 1650)	ESKD/Death (n = 431)	*p*-Value
Demographics			
Age, years (mean ± SD)	42 ± 15	45 ± 17	0.012
Age group < 40/40–64/≥65, n (%)	745 (45.2)/744 (45.1)/161 (9.8)	178 (41.4)/184 (42.8)/68 (15.8)	0.002
Weight (kg)	76 ± 16	73 ± 13	0.003
Height (cm)	167 ± 17	169 ± 10	NS
Female sex, n (%)	705 (42.7)	164 (38.1)	NS (0.080)
Clinical characteristics			
Immunosuppressive treatment, n (%)	1002 (62.9)	281 (72.6)	<0.001
Response to IST, n (%)	667 (73.5)	84 (33.5)	<0.001
Remission type—complete, n (%)	322 (48.6)	15 (18.5)	<0.001
Relapse, n (%)	206 (32.9)	41 (52.6)	0.001
Hypertension before biopsy, n (%)	559 (34.0)	177 (41.7)	0.003
Systolic BP (mmHg)	132 ± 20	139 ± 21	<0.001
Diastolic BP (mmHg)	82 ± 12	84 ± 12	0.001
Pretibial edema, n (%)	728 (47.5)	231 (56.9)	0.001
Biopsy indication, n (%)	Nephrotic syndrome: 871 (54.3%) Nephritic syndrome: 291 (18.2%) Mixed nephrotic-nephritic: 72 (4.5%) Asymptomatic urinary findings: 307 (19.2%) Other: 62 (3.9%)	Nephrotic syndrome: 163 (38.8%) Nephritic syndrome: 162 (38.6%) Mixed nephrotic-nephritic: 45 (10.7%) Asymptomatic urinary findings: 33 (7.9%) Other: 17 (4.0%)	<0.001
Diabetes mellitus, n (%)	172 (10.5)	44 (10.4)	NS (0.399)
Biochemical parameters			
Serum creatinine (mg/dL)	1.3 ± 1.3	3.2 ± 2.8	<0.001
eGFR (ml/min/1.73 m^2^, CKD-EPI 2021	87.5 ± 42.1	46.6 ± 40.8	<0.001
Urea/BUN (mg/dL)	47 ± 35	92 ± 63	<0.001
Uric acid (mg/dL)	6.3 ± 2.0	6.9 ± 1.9	<0.001
Total protein (g/dL)	6.0 ± 1.2	5.9 ± 1.0	NS
Albumin (g/dL)	3.3 ± 0.9	3.1 ± 0.7	<0.001
Total cholesterol (mg/dL)	261 ± 101	234 ± 90	<0.001
Triglycerides (mg/dL)	209 ± 130	198 ± 136	NS
HDL (mg/dL)	52 ± 21	47 ± 17	<0.001
LDL (mg/dL)	169 ± 83	152 ± 71	<0.001
Hemoglobin (g/dL)	12.9 ± 2.3	11.5 ± 2.3	<0.001
Hematocrit (%)	39 ± 6	34 ± 7	<0.001
Proteinuria (mg/day)	4735 ± 4816	4813 ± 4605	NS
Sedimentation (mm/h)	40 ± 30	52 ± 31	<0.001
Glucose (mg/dL)	98 ± 27	100 ± 29	NS
ALT (U/L)	20 ± 14	29 ± 161	0.038
Calcium (mg/dL)	8.9 ± 2.0	8.6 ± 0.8	0.005
Leukocyturia, n (%)	324 (21.0)	129 (31.6)	<0.001
Hematuria, n (%)	797 (51.0)	281 (68.2)	<0.001
ANCA positive, n (%)	32 (80.0)	60 (84.5)	NS (0.545)
Anti-PLA2R positive, n (%)	82 (23.7)	8 (18.6)	NS (0.080)
HBsAg positive, n (%)	43 (2.8)	14 (3.4)	0.009
Anti-HCV positive, n (%)	11 (0.7)	0 (0.0)	0.006
Low C3, n (%)	88 (5.8)	47 (11.5)	<0.001
Low C4, n (%)	48 (3.2)	8 (2.0)	0.021

Abbreviations: ALT, alanine aminotransferase; ANCA, antineutrophil cytoplasmic antibody; Anti-PLA2R, anti-phospholipase A_2_ receptor antibody; BUN, blood urea nitrogen; CKD-EPI, Chronic Kidney Disease Epidemiology Collaboration; ESKD, end-stage kidney disease; HBsAg, hepatitis B surface antigen; HCV, hepatitis C virus; HDL, high-density lipoprotein; IST, immunosuppressive therapy; LDL, low-density lipoprotein; NS, not significant.

**Table 4:** Histopathological findings by outcome.

Variable	Active Follow-Up (n = 1650)	ESKD/Death (n = 431)	*p*-Value
Total glomeruli, median (IQR)	15 (10–23)	14 (10–20)	0.002
Global sclerosis, median (IQR)	1 (0–3)	4 (1–6)	<0.001
Segmental sclerosis, median (IQR)	0 (0–2)	2 (0–2)	<0.001
Crescents, median (IQR)	0 (0–0)	3 (0–3)	<0.001
-Cellular crescents	0 (0–0)	2 (0–1)	<0.001
-Fibrocellular crescents	0 (0–0)	1 (0–0)	<0.001
-Fibrous crescents	0 (0–0)	0 (0–0)	<0.001
Subendothelial deposits, n (%)	78 (5.2)	61 (15.8)	<0.001
Subepithelial deposits, n (%)	183 (12.2)	49 (12.8)	NS (0.746)
Endocapillary proliferation, n (%)	215 (14.5)	104 (28.7)	<0.001
Exudative changes, n (%)	183 (12.7)	99 (28.1)	<0.001
Interstitial inflammation, n (%)	1015 (64.3)	333 (80.2)	<0.001
Interstitial fibrosis ≥25%, n (%)	199 (12.6)	136 (32.8)	<0.001
Vascular changes, n (%)	623 (39.9)	230 (56.1)	<0.001
Tubular atrophy ≥25%, n (%)	191 (11.9)	131 (31.4)	<0.001
MEST Scores			
Mesangial hypercellularity (M1), n (%)	230 (73.7)	69 (84.1)	0.049
Endocapillary hypercellularity (E1), n (%)	86 (27.8)	36 (44.4)	0.004
Segmental sclerosis (S1), n (%)	149 (47.9)	54 (65.1)	0.005
TIF (T1-T2), n (%)	169 (51.0)	66 (76.8)	<0.001
Basement membrane thickening, n (%)	673 (42.7)	140 (34.1)	0.002
IF evaluated, n (%)	1571 (97.8)	408 (96.9)	NS (0.313)
IgG deposits, positive, n (%)	591 (37.6)	131 (32.7)	NS (0.160)
IgM deposits, positive, n (%)	476 (30.5)	111 (27.8)	NS (0.583)
IgA deposits, positive, n (%)	579 (37.5)	139 (34.8)	NS (0.081)
C3 deposits, positive, n (%)	911 (59.1)	261 (63.2)	<0.001
C1q deposits, positive, n (%)	153 (9.9)	32 (8.1)	NS (0.459)
Kappa light chain deposits, n (%)	458 (31.0)	76 (21.6)	<0.001
Lambda light chain deposits, n (%)	508 (34.1)	85 (24.6)	<0.001
Electron microscopy performed, n (%)	149 (10.4)	28 (7.8)	NS (0.137)

Abbreviations: NS: not significant.

**Table 5:** Multivariable Cox proportional hazards regression for composite outcome (ESKD or death).

Variable	HR (95% CI)	*p*-Value
Age (per year)	1.00 (0.99–1.01)	0.585
Female sex (vs. male)	0.92 (0.72–1.17)	0.482
Pre-existing hypertension	0.88 (0.67–1.15)	0.347
Biopsy diagnosis (Ref: MCD)		0.012
FSGS	2.63 (1.03–6.68)	0.043
IgA nephropathy	2.33 (0.89–6.09)	0.083
Membranous nephropathy	1.44 (0.56–3.68)	0.449
MesPGN (non-IgA)	1.00 (0.19–5.27)	0.999
MPGN	2.06 (0.78–5.49)	0.147
RPGN	3.78 (1.37–10.45)	0.010
Immunosuppressive treatment (yes vs. no)	1.42 (1.06–1.90)	0.017
Hematuria (≥5 RBC/HPF)	0.76 (0.57–1.01)	0.060
Baseline eGFR (per ml/min/1.73 m^2^)	0.98 (0.98–0.98)	<0.001
Serum albumin (per g/dL)	0.65 (0.55–0.77)	<0.001
Proteinuria (per g/day)	1.03 (1.00–1.05)	0.043
Systolic blood pressure (mmHg)	1.00 (0.99–1.01)	0.640
Crescents (per glomerulus)	1.03 (0.99–1.06)	0.092
Tubular atrophy (ref: none)		0.798
Grade 1	1.10 (0.78–1.54)	0.596
Grade 2	1.27 (0.79–2.04)	0.317
Grade 3	1.19 (0.61–2.30)	0.607
Interstitial fibrosis (ref: none)		0.120
Grade 1	1.26 (0.89–1.79)	0.189
Grade 2	1.67 (1.06–2.64)	0.026
Grade 3	1.93 (0.95–3.91)	0.069
Global sclerosis (%)	1.01 (1.00–1.01)	0.015

## Data Availability

The Turkish Society of Nephrology Glomerular Diseases Working Group (TSN-GOLD) database was established on 4 April 2008, to collect data on primary glomerular diseases across Turkey. Initial results from this registry were published in 2014 and 2019, and the present manuscript reports extended outcomes from this cohort. The dataset is accessible at: http://pgh.tsn.org.tr/login.php (accessed on 9 May 2026).

## Supplementary Materials

The following supporting information can be downloaded at: https://www.mdpi.com/article/10.3390/jcm15114017/s1. Table S1. Multivariable logistic regression for composite outcome (ESKD or death); Table S2. Multivariable Cox regression for progression to ESKD (excluding deaths); Table S3. Multivariable logistic regression for ESKD (excluding deaths).

## References

[1] Boenink R., Bonthuis M., Boerstra B.A., Astley M.E., Montez de Sousa I.R., Helve J., Komissarov K.S., Comas J., Radunovic D., Buchwinkler L. (2025). The ERA Registry Annual Report 2022: Epidemiology of Kidney Replacement Therapy in Europe, with a focus on sex comparisons. Clin. Kidney J..

[2] Mandreoli M. (2023). Gender and sex in the development and progression of renal diseases. G. Di Clin. Nefrol. E Dial..

[3] Schwartzman-Morris J., Putterman C. (2012). Gender Differences in the Pathogenesis and Outcome of Lupus and of Lupus Nephritis. Clin. Dev. Immunol..

[4] Zhu X., Li H., Liu Y., You J., Qu Z., Yuan S., Peng Y., Liu F., Liu H. (2017). Tubular atrophy/interstitial fibrosis scores of Oxford classification combinded with proteinuria level at biopsy provides earlier risk prediction in lgA nephropathy. Sci. Rep..

[5] Beckwith H., Lightstone L., McAdoo S. (2022). Sex and Gender in Glomerular Disease. Semin. Nephrol..

[6] Haaskjold Y.L., Lura N.G., Bjørneklett R., Bostad L.S., Knoop T., Bostad L. (2023). Long-term follow-up of IgA nephropathy: Clinicopathological features and predictors of outcomes. Clin. Kidney J..

[7] García G.G., Iyengar A., Kaze F., Kierans C., Padilla-Altamira C., Luyckx V.A. (2022). Sex and gender differences in chronic kidney disease and access to care around the globe. Semin. Nephrol..

[8] O’Shaughnessy M.M., Hogan S.L., Thompson B.D., Coppo R., Fogo A.B., Jennette J.C. (2018). Glomerular disease frequencies by race, sex and region: Results from the International Kidney Biopsy Survey. Nephrol. Dial. Transplant..

[9] Fanouriakis A., Kostopoulou M., Cheema K., Anders H.J., Aringer M., Bajema I., Boletis J., Frangou E., A Houssiau F., Hollis J. (2020). 2019 Update of the Joint European League Against Rheumatism and European Renal Association–European Dialysis and Transplant Association (EULAR/ERA–EDTA) recommendations for the management of lupus nephritis. Ann. Rheum. Dis..

[10] Beck L.H., Ayoub I., Caster D., Choi M.J., Cobb J., Geetha D., Rheault M.N., Wadhwani S., Yau T., Whittier W.L. (2023). KDOQI US Commentary on the 2021 KDIGO Clinical Practice Guideline for the Management of Glomerular Diseases. Am. J. Kidney Dis..

[11] Zhang X., Luo F., Chen R., Shen J., Liu X., Shi Y., Yang Q., Huang T., Li H., Hu Y. (2023). Use of Histologic Parameters to Predict Glomerular Disease Progression: Findings From the China Kidney Biopsy Cohort Study. Am. J. Kidney Dis..

[12] Maksimowski N.A., Scholey J.W., Williams V.R. (2021). Sex and kidney ACE2 expression in primary focal segmental glomerulosclerosis: A NEPTUNE study. PLoS ONE.

[13] Reich H.N., Troyanov S.A.A., Scholey J.W., Cattran D.C. (2007). Remission of Proteinuria Improves Prognosis in IgA Nephropathy. J. Am. Soc. Nephrol..

[14] Barbour S.J., Coppo R., Zhang H., Liu Z.H., Suzuki Y., Matsuzaki K., Katafuchi R., Er L., Espino-Hernandez G., Kim S.J. (2019). Evaluating a New International Risk-Prediction Tool in IgA Nephropathy. JAMA Intern. Med..

[15] Iwasaki C., Moriyama T., Tanaka K., Takei T., Nitta K. (2016). Effect of hematuria on the outcome of immunoglobulin A nephropathy with proteinuria. J. Nephropathol..

[16] Wang R., Zhang X., Wang X., Chen L., Ma Q., Su Y., Liu J., Shi H. (2023). Evaluation of three scoring systems for predicting renal prognosis in antineutrophil cytoplasmic antibody-associated glomerulonephritis. Eur. J. Med. Res..

[17] Mohamed O.N., Ibrahim S.A., Saleh R.K., Issa A.S., Setouhi A., Rabou A.A.A., Mohamed M.R., Kamel S.F. (2024). Clinicopathological characteristics and predictors of outcome of rapidly progressive glomerulonephritis: A retrospective study. BMC Nephrol..

[18] Wilson G.J., Cho Y., Teixiera-Pinto A., Isbel N., Campbell S., Hawley C., Johnson D.W. (2019). Long-term outcomes of patients with end-stage kidney disease due to membranoproliferative glomerulonephritis: An ANZDATA registry study. BMC Nephrol..

[19] Zhao H., Li Y., Sun J., Xu G., Wang C., Zhou S., Nie S., Li Y., Su L., Chen R. (2023). Immunosuppression versus Supportive Care on Kidney Outcomes in IgA Nephropathy in the Real-World Setting. Clin. J. Am. Soc. Nephrol..

[20] Blazius B., Troost J.P., Kopp J.B., Parekh R.S., Gillespie B., Ayoub I., Kallash M., Gbadegesin R., Canetta P.A., Srivastava T. (2025). Clinical Decision-Making About Immunosuppressive Treatment in Focal Segmental Glomerulosclerosis. Kidney Med..

[21] Rauen T., Eitner F., Fitzner C., Sommerer C., Zeier M., Otte B., Panzer U., Peters H., Benck U., Mertens P.R. (2015). Intensive Supportive Care plus Immunosuppression in IgA Nephropathy. N. Engl. J. Med..

