# Supplementary material for: Sex Differences in Baseline Characteristics and Long-Term Outcomes of Primary Glomerular Diseases: Insights from the TSN-GOLD Registry
Source: J Clin Med. 2026 May 22;15(11):4017. doi: 10.3390/jcm15114017 (PMC13257337; doi:10.3390/jcm15114017)
Supplement: Supplementary file 1 [file jcm-15-04017-s001.zip › jcm-4226606-supplementary.pdf]

Suppl. Table S1. Multivariable logistic regression for composite outcome (ESKD or death)

**Variable**	**OR (95% CI)**	**p-value**
Age (per year)	0.99 (0.98 - 1.00)	0.193
Female sex (vs male)	0.80 (0.59 - 1.08)	0.138
**Biopsy diagnosis (Ref: MCD)**		0.002
- FSGS	2.41 (0.95 - 6.13)	0.064
- IgA nephropathy	2.35 (0.90 - 6.16)	0.082
- Membranous nephropathy	1.74 (0.69 - 4.40)	0.239
- MesPGN (non-IgA)	1.09 (0.19 - 6.22)	0.921
- MPGN	3.13 (1.16 - 8.47)	0.024
- RPGN	5.65 (2.06 - 15.51)	0.001
Immunosuppressive treatment (yes vs no)	1.05 (0.75 - 1.48)	0.760
Pre-existing hypertension	0.89 (0.65 - 1.24)	0.500
Hematuria (≥5 RBC/HPF)	0.75 (0.55 - 1.04)	0.081
Hemoglobin (g/dl)	0.94 (0.88 - 1.01)	0.092
Proteinuria (per g/day)	1.00 (0.97 - 1.04)	0.882
Serum albumin (per g/dl)	0.64 (0.52 - 0.80)	<0.001
Systolic blood pressure (mmHg)	1.01 (0.99 - 1.01)	0.133
Baseline eGFR (per ml/min/1.73 m^2)	0.98 (0.98 - 0.99)	<0.001
Tubular atrophy (ref: none)		0.012
- Grade 1	0.97 (0.68 - 1.37)	0.842
- Grade 2	1.50 (0.94 - 2.40)	0.087

- Grade 3	2.50 (1.26 - 4.94)	0.009
Interstitial inflammation (yes vs no)	1.33 (0.92 - 1.90)	0.127
Global sclerosis (%)	1.01 (1.00 - 1.01)	0.188

Suppl Table S2. Multivariable Cox regression for progression to ESKD (excluding deaths)

Variable	HR (95% CI)	p-value
**Age (per year)**	0.98 (0.96 - 0.99)	<0.001
**Female sex (vs male)**	0.89 (0.65 - 1.20)	0.435
**Pre-existing hypertension**	0.95 (0.68 - 1.32)	0.759
**Biopsy diagnosis (Ref: MCD)**		0.006
**- FSGS**	2.78 (0.65 - 11.82)	0.166
**- IgA nephropathy**	2.92 (0.67 - 12.67)	0.152
**- Membranous nephropathy**	1.20 (0.27 - 5.35)	0.816
**- MesPGN (non-IgA)**	1.59 (0.22 - 11.76)	0.650
**- MPGN**	2.69 (0.61 - 11.88)	0.192
**- RPGN**	5.78 (1.27 - 26.33)	0.023
**Immunosuppressive treatment (yes vs no)**	1.71 (1.22 - 2.40)	0.002
**Hematuria (≥5 RBC/HPF)**	0.65 (0.45 - 0.93)	0.018
**Baseline eGFR (per ml/min/1.73 m²)**	0.97 (0.96 - 0.97)	<0.001
**Serum albumin (per g/dl)**	0.68 (0.55 - 0.83)	<0.001
**Proteinuria (per g/day)**	1.05 (1.02 - 1.08)	0.001
**Systolic blood pressure (mmHg)**	1.00 (1.00 - 1.01)	0.436
**Crescents (per glomerulus)**	1.00 (0.96 - 1.04)	0.918
**Tubular atrophy (ref: none)**		0.371
**- Grade 1**	1.30 (0.84 - 2.02)	0.240
**- Grade 2**	1.13 (0.62 - 2.07)	0.692

**- Grade 3**	0.75 (0.34 - 1.64)	0.466
**Interstitial fibrosis (ref: none)**		0.018
**- Grade 1**	1.39 (0.88 - 2.21)	0.163
**- Grade 2**	2.36 (1.32 - 4.21)	0.004
**- Grade 3**	3.02 (1.31 - 6.94)	0.009
**Global sclerosis (%)**	1.01 (1.00 - 1.01)	0.099

Suppl Table S3. Multivariable logistic regression for ESKD (excluding deaths)

Variable	OR (95% CI)	p-value
**Age (per year)**	0.99 (0.98 - 1.00)	0.193
**Female sex (vs male)**	0.80 (0.59 - 1.08)	0.138
**Pre-existing hypertension**	0.89 (0.65 - 1.24)	0.500
**Biopsy diagnosis (Ref: MCD)**		0.002
**- FSGS**	2.41 (0.95 - 6.13)	0.064
**- IgA nephropathy**	2.35 (0.90 - 6.16)	0.082
**- Membranous nephropathy**	1.74 (0.69 - 4.40)	0.239
**- MesPGN (non-IgA)**	1.09 (0.19 - 6.22)	0.921
**- MPGN**	3.13 (1.16 - 8.47)	0.024
**- RPGN**	5.65 (2.06 - 15.51)	0.001
**Immunosuppressive treatment**	1.05 (0.75 - 1.48)	0.760
**Hematuria (≥5 RBC/HPF)**	0.76 (0.55 - 1.04)	0.081
**Hemoglobin (per g/dl)**	0.94 (0.88 - 1.01)	0.092
**Proteinuria (per g/day)**	1.00 (0.97 - 1.04)	0.882
**Albumin (per g/dl)**	0.64 (0.52 - 0.80)	<0.001
**Systolic BP (mmHg)**	1.01 (1.00 - 1.01)	0.133
**Baseline eGFR (per ml/min/1.73 m^2)**	0.98 (0.98 - 0.99)	<0.001
**Tubular atrophy (ref: none)**		0.012
**- Grade 1**	0.97 (0.68 - 1.37)	0.842
**- Grade 2**	1.50 (0.94 - 2.40)	0.087

**- Grade 3**	2.50 (1.26 - 4.94)	0.009
**Interstitial inflammation**	1.33 (0.92 - 1.90)	0.127
**Global sclerosis (%)**	1.01 (1.00 - 1.01)	0.188
